# Association between infant feeding patterns and diarrhoeal and respiratory illness: A cohort study in Chittagong, Bangladesh

**DOI:** 10.1186/1746-4358-3-28

**Published:** 2008-11-24

**Authors:** Seema Mihrshahi, Wendy H Oddy, Jennifer K Peat, Iqbal Kabir

**Affiliations:** 1 Department of Medicine, University of Melbourne, The Royal Melbourne Hospital, Parkville, Victoria, Australia; 2 The Telethon Institute for Child Health, Centre for Child Health Research and University of Western Australia, Western Australia, Australia; 3 Independent Research Consultant, Tomerong, New South Wales, Australia; 4 International Centre for Diarrhoeal Diseases Research, Bangladesh

## Abstract

**Background:**

In developing countries, infectious diseases such as diarrhoea and acute respiratory infections are the main cause of mortality and morbidity in infants aged less than one year. The importance of exclusive breastfeeding in the prevention of infectious diseases during infancy is well known. Although breastfeeding is almost universal in Bangladesh, the rates of exclusive breastfeeding remain low. This cohort study was designed to compare the prevalence of diarrhoea and acute respiratory infection (ARI) in infants according to their breastfeeding status in a prospective cohort of infants from birth to six months of age.

**Methods:**

A total of 351 pregnant women were recruited in the Anowara subdistrict of Chittagong. Breastfeeding practices and the 7-day prevalence of diarrhoea and ARI were recorded at monthly home visits. Prevalences were compared using chi-squared tests and logistic regression.

**Results:**

A total of 272 mother-infant pairs completed the study to six months. Infants who were exclusively breastfed for six months had a significantly lower 7-day prevalence of diarrhoea [AOR for lack of EBF = 2.50 (95%CI 1.10, 5.69), p = 0.03] and a significantly lower 7-day prevalence of ARI [AOR for lack of EBF = 2.31 (95%CI 1.33, 4.00), p < 0.01] than infants who were not exclusively breastfed. However, when the association between patterns of infant feeding (exclusive, predominant and partial breastfeeding) and illness was investigated in more detail, there was no significant difference in the prevalence of diarrhoea between exclusively [6.6% (95% CI 2.8, 10.4)] and predominantly breastfed infants [3.7% (95% CI 0.09, 18.3), (p = 0.56)]. Partially breastfed infants had a higher prevalence of diarrhoea than the others [19.2% (95% CI 10.4, 27.9), (p = 0.01)]. Similarly, although there was a large difference in prevalence in acute respiratory illness between exclusively [54.2% (95%CI 46.6, 61.8)] and predominantly breastfed infants [70.4% (95%CI 53.2, 87.6)] there was no significant difference in the prevalence (p = 0.17).

**Conclusion:**

The findings suggest that exclusive or predominant breastfeeding can reduce rates of morbidity significantly in this region of rural Bangladesh.

## Background

In Bangladesh, infectious diseases such as diarrhoea and acute respiratory infections are the main cause of mortality and morbidity in infants aged less than one year [1,2]. The importance of breastfeeding in the prevention of infectious diseases during infancy is well known [3,4]. Although breastfeeding is almost universal in Bangladesh, the rates of exclusive breastfeeding remain low. Recent data show that only 38% of children aged 2–3 months are exclusively breastfed and 23% of children are given complementary foods before the sixth month [2]. Previous studies conducted in urban areas of Bangladesh using individual peer counseling have shown significant increases in the rates of exclusive breastfeeding [5]. The Chittagong peer counseling study was a randomized trial undertaken to assess the impact of group and individual peer counseling on exclusive breastfeeding rates in Anowara, a rural area of Chittagong district [6]. The study was conducted by staff from International Centre for Diarrhoeal Diseases Research, Bangladesh (ICDDR, B) from 1999 to 2001. The peer counseling study was designed to compare the effectiveness of individual versus group counseling in achieving improved exclusive breastfeeding rates. This paper presents the results of a secondary analysis of the data where the data were analyzed without regard to randomization group. The main aim of this analysis was to measure the association between exclusive breastfeeding and infectious disease (diarrhoea and acute respiratory illness) in a cohort of women and children followed from birth until six months of age.

## Methods

### Study design and setting

The study was conducted from September 1999 to March 2001 in three unions of Anowara thana (sub district) in Chittagong district, a rural area in south eastern Bangladesh approximately 40 km from Chittagong City.

The main study was a randomized controlled intervention trial where pregnant women in two of the unions were randomized to the intervention (group or individual peer counseling) and one union was used as a control group. The details of this study including the sample size and CONSORT diagram have been presented [6]. For the purposes of this analysis, the data are analyzed as a cohort study with infants followed up from birth to six months of age.

### Recruitment and inclusion and exclusion criteria

**Figure 1:**
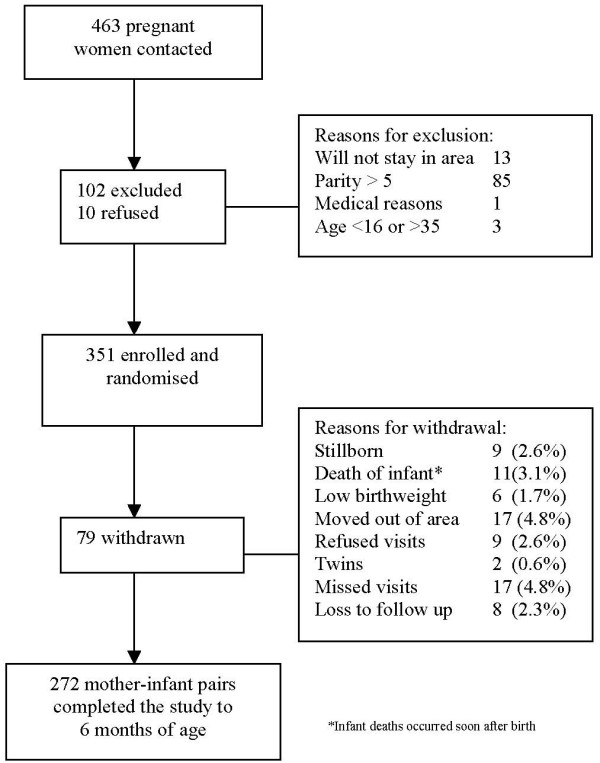
Chittagong cohort study trial profile.

A total of 351 pregnant women were recruited and 272 mother infant pairs completed the study to six months (Figure 1). Pregnant women aged 16–35 years, with no more than three living children or a parity of five, who intended to deliver and stay in the study area were asked to participate. Women with documented heart disease, insulin dependent diabetes mellitus, or eclampsia during the previous pregnancy were excluded. Infants were excluded from the study if they had congenital abnormalities; were admitted to intensive care after birth; were twins/triplets or had weight below 1800 g on the fourth day after birth.

### Data collection and definitions

An interviewer visited each mother-infant pair at home eight times during the study period. Data on socioeconomic, demographic and environmental variables, maternal and pregnancy factors, and previous infant feeding practices were obtained at enrolment. Details of delivery and early infant feeding were collected on the fourth day after birth. Thereafter, information on infant feeding and illness was obtained monthly until the infant was six months of age.

At each home visit, infant feeding status was assessed using 24 hour recall. In addition to this the mother was asked if she had fed her baby anything other than breast milk since the last visit. If she had fed anything else on two successive days or more, the information was taken into consideration when classifying the feeding status as exclusive, predominant, partial or no breastfeeding. Definitions used in the study:

• *Exclusive breastfeeding *was defined when the infant received breast milk only and no other solids or liquids with the exception of vitamins, minerals, medicines or oral rehydration solution;

• *Predominant breastfeeding *was defined when the infant received breast milk and water, water based liquids such as sugar water and juices but not infant formula or milk;

• *Partial breastfeeding *was defined when the infant received breast milk in addition to complementary foods;

• *Non-breastfed *was when the infant received no breast milk;

• *Complementary foods *included milk, infant formula, gruel or semi-solid foods given in addition to breast milk;

• *Prelacteal foods *refer to non-breast milk feeds given before breastfeeding is initiated such as honey and mustard oil.

Definitions of primary outcomes:

• *Diarrhoea *was defined as three or more loose stools in 24 hours in the past week (seven days) at any monthly visit during the first six months of life;

• *Acute respiratory infection *was defined as cough and fever in the past week (seven days) at any monthly visit during the first six months of life.

### Ethics approval

Ethical approval for the randomized trial was obtained from the Ethical Review Committee of the ICDDR, B. Signed, informed consent was obtained from each mother and her husband or guardian. Ethics approval for the secondary analysis was obtained by Human Research Ethics Committee of Curtin University and ICDDR, B.

### Data analyses

Statistical analyses were conducted using SPSS (version 13.0). The primary outcome variables were the 7-day prevalence of diarrhoea and acute respiratory illness at any monthly visit in the first six months of life (ie. analysis was conducted by infant and not by number of episodes). Explanatory variables were exclusive, predominant, partial and no breastfeeding, categorized at the six month visit. The breastfeeding variable was also dichotomized as exclusive breastfeeding or not exclusive breastfeeding (which included predominant, partial and no breastfeeding) at the six month visit. Chi-squared tests and logistic regression were used to test associations. Core confounders such as infant's gender, mother's education and work status, number of siblings, latrine type, assets and drinking water source were left in the final model. Other possible confounders such as mother's age, father's education and occupation, house type, house ownership, and mother's height and weight and intervention group were included in the model if they changed the standard error by 5% or more. Results are presented as odds ratios and adjusted odds ratios.

## Results

A total of 463 pregnant women were contacted for entry into the trial and 122 women fulfilled the exclusion criteria. A total of 351 women were enrolled and randomized into the main trial. Of these women, 272 (77.5%) completed the study to six months. Figure 1 shows the trial profile and reasons for exclusion and loss to follow up.

### Baseline characteristics of the study population

Table 1 shows the baseline characteristics in families who completed the study to six months. The mean number of members in each household was 5.64 (SD 3.31). A total of 53.7% of households had more than five members. Mean age of the women was 22.63 years (SD 4.4). On average men went to school for longer than women and most were in some form of paid employment. A majority of families (84%) owned their own dwelling and had access to a latrine which was shared with other families.

**Table 1:** Baseline characteristics of families in the study (N = 272)

**Characteristic**		**n**	**%**
Total household members	1–2	17	6.3
	3–4	109	40.1
	5 or more	146	53.6

Husband resides	In same house	209	76.8
	Separated	3	1.1
	Lives abroad	29	10.7
	Comes and goes	31	10.4

Mother's age	16–20	101	37.1
	21–25	106	39.0
	26–30	49	18.0
	31–35	16	5.9

Parity	One	79	29.0
	Two	69	25.4
	Three or more	124	45.6

Antenatal care	Yes	109	40.1
	No	163	59.9

Mother's education	None	125	46.0
	Primary	60	22.0
	Secondary and above	87	32.0

Father's education	None	96	35.3
	Primary	77	28.3
	Secondary and above	99	36.4

There were some differences between families in the study and those who were withdrawn (n = 79). Families who completed the study were more likely to have husbands/fathers who lived at home than families who withdrew (77% vs 65%, p = 0.03). Families who completed the study to six months were also more likely to have other children (66% vs 48%, p < 0.01) and more children in the household in total than families who withdrew (chi-square for trend = 6.757, p < 0.01). However, the overall number of household members was comparable between groups. Families who completed the study to six months were more likely to own their own house than those who were withdrawn (89% vs 81%, p = 0.049). There were no differences in maternal weight, height or underarm circumference, education status, occupation or household income between the groups.

Table 2 shows the delivery and birth characteristics of the babies in the study and those babies who were withdrawn or lost to follow up. A total of nine babies were stillborn. The gestational age was not different between groups. However, babies' weight on day four was significantly less in the group who withdrew (p < 0.01) as was the length (p < 0.01). Most babies were delivered vaginally, at home, by untrained dais or birth attendants. There were no differences in delivery characteristics or babies' gender between groups.

**Table 2:** Comparison of delivery characteristics between infants in study and those who were withdrawn

	**Completed study**		**Withdrawn**	
	**N**	**Mean (SD) or n (%)**	**N**	**Mean (SD) or n (%)**
**Gestational age (weeks)** Mean (SD)	272	39.1 (2.8)	70	39.5 (3.4)
**Weight on day 4 (kg)** Mean (SD)	246	2.7 (0.4)	51	2.5 (0.5)
**Length on day 4 (cm)** Mean (SD)	244	47.5 (1.9)	51	46.6 (2.9)
**Place of delivery**	272		70	
home		240 (88.2%)		63 (90%)
hospital/clinic		32 (11.8%)		7 (10%)
**Type of delivery**	272		70	
normal vaginal		268 (98.5%)		67 (95.7%)
vaginal assisted		-		1 (1.4%)
caesarean section		4 (1.5%)		2 (2.9%)
**Baby delivered by**	272		70	
trained birth attendant		26 (9.6%)		4 (5.7%)
untrained attendant		155 (57.0%)		36 (51.4%)
experienced relative		43 (15.8%)		15 (21.4%)
nurse or doctor		12 (4.4%)		10 (14.3%)
family welfare visitor		29 (10.7%)		5 (7.1)
other		7 (2.6%)		-
**Baby's gender**	272		70	
male		127 (46.7%)		37 (52.9%)
female		145 (53.3%)		33 (47.1%)

Values are mean (SD), number (%)

### Infant feeding practices

Table 3 shows the early feeding practices of the infants in the cohort who completed the study. All babies were fed colostrum, therefore breastfeeding was initiated within three days of birth in all cases and commonly within three hours of birth. However, in 70% of cases colostrum was not the first food given to the child and other prelacteal foods were given, the most common being mustard oil or honey. The most common reason for feeding of prelacteal foods was that it was a custom or tradition to do so.

**Table 3:** Early feeding practices in Chittagong cohort (N = 272)

	**n**	**%**
**First food given to child**		
colostrum	80	29.4
honey	45	16.5
misri/sugar water	8	2.9
honey + mustard oil	2	0.7
water	19	7.0
mustard oil	82	30.1
powder milk	2	0.7
other	34	12.5

Reason for feeding prelacteal foods*		
sweet voice	3	1.6
satisfy hunger	13	6.8
prevent cold	3	1.6
clean stomach/mouth/throat	54	28.1
tradition	116	60.4
stop baby crying	2	1.0
colostrum insufficient	1	0.5
mother sick	5	2.6
baby didn't suckle	3	1.6
other	4	2.1

**Initiation of breastfeeding**		
within 1 hr	62	22.8
within 2 hrs	82	30.1
within 3 hrs	17	6.3
4–6 hrs	31	11.4
7–12 hrs	30	11.0
12–24 hrs	18	6.6
>24 hrs	30	11.0
missing	2	0.7

*Sample size for this variable was 192

**Figure 2:**
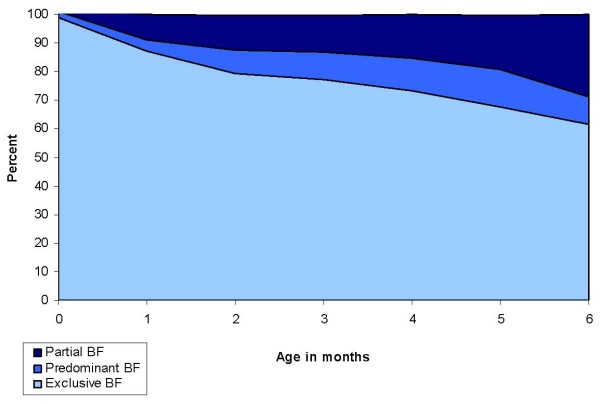
Distribution of breastfeeding (BF) patterns by age in the Chittagong Cohort Study.

Figure 2 shows the distribution of breastfeeding patterns by age of the child. If prelacteal foods were excluded from the analysis, there was a high rate of exclusive breastfeeding throughout the study with 87.1% of mothers exclusively breastfeeding at one month. The rate of exclusive breastfeeding gradually declined to 77.2% at three months and 61.4% at six months. Because of the rigorous methods of classification, once a woman was categorised in the predominant breastfeeding category she was not reclassified as exclusively breastfeeding and similarly if a woman was categorised as partially breastfeeding she was not reclassified as predominantly breastfeeding at subsequent visits. At the six month visit a total of 167 (61.4%) of infants were exclusively breastfed, 27 (9.9%) infants were predominantly breastfed and 77 (28.3%) infants were partially breastfed. Only one child was not breastfed in the entire cohort.

### Prevalence of illness episodes

Table 4 shows the numbers of illness episodes in the previous seven days at each visit. The 7-day cumulative prevalence of diarrhoea at each monthly visit in the first six months was 9.9% (27/272), of ARI was 61.7% (168/272) and of any illness was 95.6% (260/272).

**Table 4:** Episodes of illness and prevalence in last 7-days at monthly visits in the first six months of life, n (%)

**Visit**	**Diarrhoea**	**Cough**	**Fever**	ARI*	**Any illness**
1 month	4 (1.5)	130 (47.8)	50 (18.4)	41 (15.0)	157 (57.7)
2 month	2 (0.7)	103 (37.9)	57 (21.0)	46 (16.9)	132 (48.5)
3 month	4 (1.5)	78 (28.7)	46 (16.9)	38 (14.0)	99 (36.4)
4 month	4 (1.5)	83 (30.5)	52 (19.1)	45 (16.5)	106 (39.0)
5 month	7 (2.6)	94 (34.6)	68 (25.0)	59 (21.7)	123 (45.2)
6 month	9 (3.3)	101 (37.1)	82 (30.1)	63 (23.2)	149 (54.8)

*Acute respiratory infection

### Prevalence of illness between exclusively breastfed and non-exclusively breastfed infants and association with infant feeding patterns

**Figure 3:**
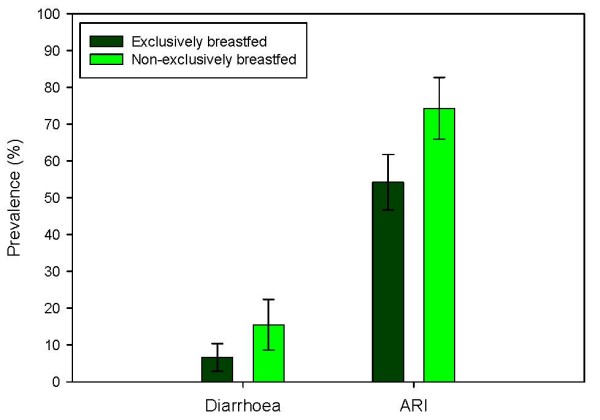
Prevalence of diarrhoea and acute respiratory infections (ARI) between infants exclusively breastfed and infants non-exclusively breastfed in the Chittagong cohort study (error bars represent 95% confidence intervals).

Figure 3 shows the difference in prevalence of diarrhoea and acute respiratory infection between infants who were and who were not exclusively breastfed for at least six months. The group who were exclusively breastfed for six months had an 8.6% lower 7-day prevalence of diarrhoea and a 20% lower 7-day prevalence of ARI than the group who were not exclusively breastfed. There was a significant association between the lack of exclusive breastfeeding and diarrhoea and acute respiratory infection in the groups. These significant differences remained even after adjustment for mother's education, asset score and husband living at home as a measure of socioeconomic status, type of latrine as a measure of sanitation, gender and other siblings. The adjusted odds ratio for diarrhoea is 2.50 (95% CI 1.10, 5.69) and for acute respiratory infection is 2.31 (95% CI 1.33, 4.00).

**Figure 4:**
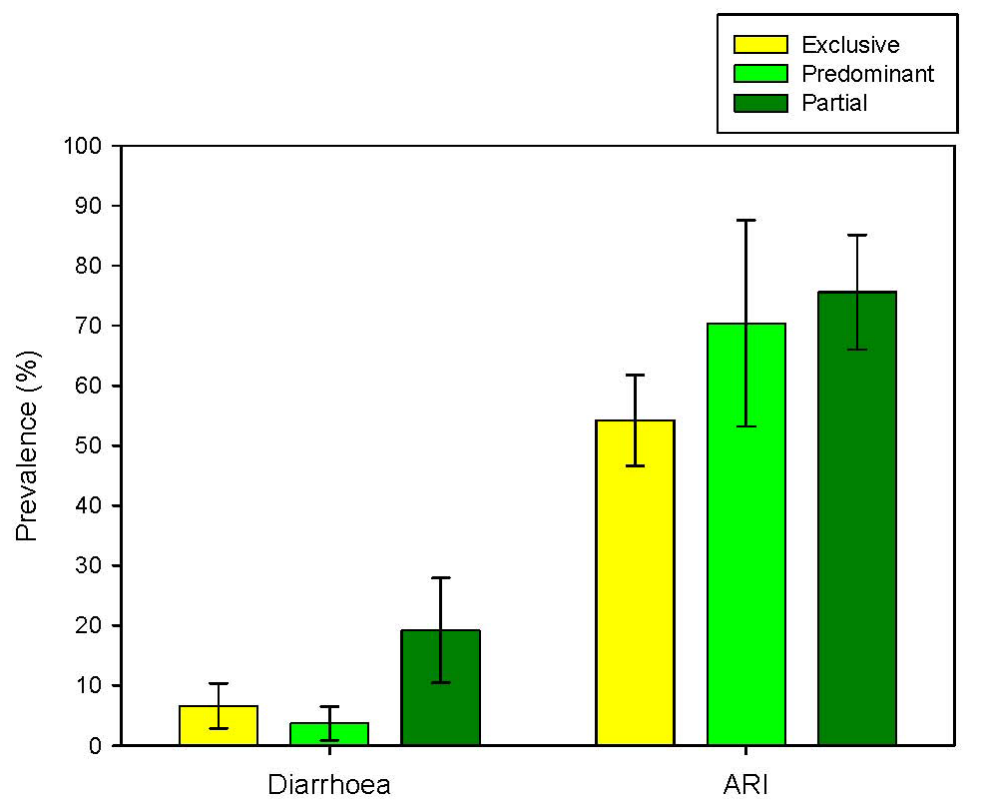
Prevalence of diarrhoea and acute respiratory infections (ARI) between infants according to patterns of breastfeeding in the Chittagong cohort study (error bars represent 95% confidence intervals).

The association between patterns of infant feeding (exclusive, predominant and partial breastfeeding at six months) and illness was investigated, and the prevalences are shown in Figure 4. The number of predominantly breastfed infants was low (n = 27) and hence the confidence intervals are large. There was no significant difference in the prevalence of diarrhoea between exclusively [Diarrhoea prevalence 6.6% (95% CI 2.8, 10.4)] and predominantly breastfed infants [Diarrhoea prevalence 3.7% (95% CI 0.09, 18.3), (p = 0.56)], however partially breastfed infants had a higher prevalence of diarrhoea than the others [Diarrhoea prevalence 19.2% (95% CI 10.4, 27.9), (p = 0.01)]. Similarly, although there was a large difference in prevalence in acute respiratory illness between exclusively [ARI prevalence 54.2% (95%CI 46.6, 61.8)] and predominantly breastfed infants [ARI prevalence 70.4% (95%CI 53.2, 87.6)] there was no significant difference in the prevalence (p = 0.17). Partially breastfed infants had a significantly higher prevalence [ARI prevalence 77.4% (95%CI 67.3, 85.0)] and the data showed a significant trend of breastfeeding patterns on ARI (p value for trend < 0.01). Combining the exposure and outcome into three monthly observations to avoid misclassification showed similar results (data not shown).

The multivariate analysis showed similar results. Exclusive breastfeeding was not significantly more protective than predominant breastfeeding for diarrhoeal illness but was significantly more protective than partial breastfeeding. Similarly exclusive breastfeeding was not significantly more protective than predominant breastfeeding for acute respiratory infection but was significantly more protective than partial breastfeeding (Table 5).

**Table 5:** Association between patterns of breastfeeding and diarrhoea and acute respiratory infection in the Chittagong cohort

**Breastfeeding practice**	**Diarrhoea**		**Acute respiratory infections**	
	
	AOR* (95%CI)	**p value**	AOR* (95%CI)	**p value**
Exclusive (n = 167)	1.00		1.00	

Predominant (n = 27)	0.48 (0.06, 3.95)	0.50	1.85 (0.76, 4.69)	0.17

Partial (n = 78)	3.43 (1.47, 8.03)	<0.01	2.49 (1.34, 4.63)	<0.01

* Adjusted Odds Ratio: adjusted for mother's education, asset score and husband living with family as a measure of socioeconomic status, type of latrine as a measure of sanitation, gender, other siblings.

## Discussion

This study has shown that in a cohort of infants in rural Bangladesh followed up from birth to six months of age, infants who were exclusively breastfed for six months had a significantly lower 7-day prevalence of diarrhoea [AOR for lack of EBF = 2.50 (95% CI 1.10, 5.69), p = 0.03] and a significantly lower 7-day prevalence of ARI [AOR for lack of EBF = 2.31 (95% CI 1.33, 4.00), p < 0.01] than infants who were not exclusively breastfed. This effect was significant and remained even after controlling for several potential confounders. However, when the association between patterns of infant feeding (exclusive, predominant and partial breastfeeding) and illness was investigated in more detail, exclusive breastfeeding was not significantly more protective than predominant breastfeeding for preventing diarrhoeal illness although it was significantly more protective than partial breastfeeding. Similarly, exclusive breastfeeding was not significantly more protective than predominant breastfeeding for preventing acute respiratory infection but it was significantly more protective than partial breastfeeding. Partially breastfed infants had other sources of nutrition than breastmilk, with infant formula, other liquids, milks and solid foods part of their regular diet. These findings suggest that predominant breastfeeding may be sufficient to reduce rates of morbidity significantly in this rural area of Bangladesh. However, the number of predominantly breastfed infants was small (n = 27) and further studies with larger numbers would be needed to be certain that there are no differences in these two infectious disease outcomes between exclusively and predominantly breastfed infants.

In a recent study with data from India, Peru and Ghana a similar effect was shown on hospitalizations for diarrhoeal and respiratory illness [7]. There was no significant difference in the risk of hospitalization between infants who were exclusively breastfed compared with infants who were predominantly breastfed [adjusted rate ratio = 0.67 (95% CI 0.23, 2.01)]. However, non-breastfed infants had a higher risk of all cause hospitalization when compared with infants who had been predominantly breastfed [adjusted rate ratio = 3.39 (95% CI 1.74, 6.61); p < 0.01] and also had a higher diarrhoea specific hospitalization [adjusted rate ratio = 5.59 (95% CI 2.17, 14.4); p < 0.01]. A similar effect was seen on mortality with no significant difference in the risk of death between children who were exclusively breastfed and those who were predominantly breastfed [adjusted hazard ratio = 1.46 (95% CI 0.75, 2.86)]. However, non breastfed infants had a higher risk of dying when compared with infants who had been predominantly breastfed [adjusted hazard ratio = 2.46 (95% CI 1.44, 4.18)]; p < 0.01]. This led the authors to conclude that where rates of predominant breastfeeding are already high, promotion efforts should focus on sustaining these high rates rather than on attempting to achieve a shift from predominant breastfeeding to exclusive breastfeeding [7].

One of the major problems when comparing similarly designed studies in different settings is that infant feeding patterns are not well defined. An advantage in this study was that four levels of infant feeding pattern could be compared. These were non breastfed, partially breastfed, predominantly breastfed and exclusively breastfed and WHO definitions of exclusive and predominant breastfeeding [8] were applied. However, due to the very small number of infants that didn't breastfeed, there was no non breastfed category for comparison in our study. In some studies the categories of partial and predominantly breastfed are merged into a 'mixed fed' category [9,10]. Other studies compare any breastfeeding with non breastfed [11], or compare exclusively breastfed infants with non exclusively breastfed infants [12] making it difficult to compare results of all studies according to outcomes.

There were no differences in morbidity patterns between infants who were exclusively breastfed and those who were predominantly breastfed (ie breastmilk and water based drinks but no infant formula/milk or solid food). It may be that in certain settings where purity of drinking water is good and standards of hygiene are high, predominant breastfeeding may be as safe as exclusive breastfeeding for the infant. However in countries such as Bangladesh, especially in rural areas these conditions are unlikely, so the results of this study are surprising. Some studies suggest that predominantly breastfed infants who have had tastes of water and honey have had damage to the mucosal lining of gastrointestinal tract leaving the infant more susceptible to infection [13], but this effect could not be confirmed in this cohort.

A limitation of this cohort study is that the definition of exclusive breastfeeding included some of those infants who had received prelacteal feeds, as long as the feeds were not given two successive days or more. If truly exclusively breastfed infants (ie. exclusively breastfed since birth) were compared to predominately breastfed infants, larger differences in infectious disease may have been seen. Another limitation was the differences between families who remained in the study for the six month period and those that withdrew. Families who stayed in the study were more likely to have a stable family structure (ie. husbands living at home, and owning their own home). They were more likely to have other children and a larger number of children although the overall family size, and therefore the likelihood of crowding, was similar. These factors are unlikely to have added considerable bias to the study results. There was no difference in gestational age or other characteristics between groups although the babies in the group that withdrew had a lower weight and length than those who stayed in the study. This may be an indication that the analysis included infants who were healthier.

Misclassification of the exposure was also a possibility. The category of breastfeeding (exclusive, predominant or partial) was classified at the six month visit while the outcome was measured at monthly visits to avoid recall bias. In order to minimize misclassification the outcomes and exposures in the first three months and last three months were combined and the results were similar to the grouped analysis. That is, that exclusive breastfeeding was not significantly more protective than predominant breastfeeding for preventing diarrhoeal illness or ARI although it was significantly more protective than partial breastfeeding.

The problem of reverse causality may be a limitation to this study. For example, if mothers tended to breastfeed exclusively because the child was ill, the effect of exclusive breastfeeding on illness would have been underestimated. Conversely, if mothers stopped breastfeeding as a result of illness this would have biased the results towards an overestimation of an effect. A way to minimize this would be to ascertain whether any of the mothers changed their breastfeeding behavior as a result of illness. Because of the generally high rates of continued exclusive breastfeeding, the assumption is that this would lead to an underestimation of any association with respect to illness.

Another limitation to the study is the high number of stillborn babies (9/351 or 2.6%). This figure is typical of the rural population of Bangladesh where most women do not have access to antenatal care and almost 90% of women deliver at home without specialized delivery care.

The rates of prelacteal feeding and reasons for prelacteal feeding are consistent with other descriptive studies undertaken in rural Bangladesh [14,15]. There was a high rate of exclusive breastfeeding throughout the study with 87.1% of mothers exclusively breastfeeding at one month. The rate of exclusive breastfeeding gradually declined to 77.2% at three months and 61.4% at six months. Only one child in the whole cohort was not breastfed.

## Conclusion

In summary, this cohort study conducted in Chittagong in Bangladesh has shown that infants who are exclusively breastfed from birth to six months of age have a significantly lower prevalence of diarrhoea and acute respiratory infection than those infants who are not exclusively breastfed. However, there were no differences in morbidity prevalence between infants who were exclusively breastfed or predominantly breastfed. The results of this study seem to suggest that both exclusive and predominant breastfeeding can reduce morbidity in this rural area of Bangladesh.

## Competing interests

The authors declare that they have no competing interests.

## Authors' contributions

IK was responsible for the study design, conduct and data collection, SM performed the data analysis, interpreted the data and prepared the manuscript. WO and JKP provided content advice, interpretation of data and statistical advice for the manuscript. All authors read and approved the final manuscript.
